# Atomistic mechanism of transmembrane helix association

**DOI:** 10.1371/journal.pcbi.1007919

**Published:** 2020-06-04

**Authors:** Jan Domański, Mark S. P. Sansom, Phillip J. Stansfeld, Robert B. Best

**Affiliations:** 1 Laboratory of Chemical Physics, National Institute of Diabetes and Digestive and Kidney Diseases, National Institutes of Health, Bethesda, Maryland, United States of America; 2 Department of Biochemistry, University of Oxford, South Parks Road, Oxford, United Kingdom; 3 School of Life Sciences and Department of Chemistry, University of Warwick, Gibbet Hill Campus, Coventry, United Kingdom; University of Virginia, UNITED STATES

## Abstract

Transmembrane helix association is a fundamental step in the folding of helical membrane proteins. The prototypical example of this association is formation of the glycophorin dimer. While its structure and stability have been well-characterized experimentally, the detailed assembly mechanism is harder to obtain. Here, we use all-atom simulations within phospholipid membrane to study glycophorin association. We find that initial association results in the formation of a non-native intermediate, separated by a significant free energy barrier from the dimer with a native binding interface. We have used transition-path sampling to determine the association mechanism. We find that the mechanism of the initial bimolecular association to form the intermediate state can be mediated by many possible contacts, but seems to be particularly favoured by formation of non-native contacts between the C-termini of the two helices. On the other hand, the contacts which are key to determining progression from the intermediate to the native state are those which define the native binding interface, reminiscent of the role played by native contacts in determining folding of globular proteins. As a check on the simulations, we have computed association and dissociation rates from the transition-path sampling. We obtain results in reasonable accord with available experimental data, after correcting for differences in native state stability. Our results yield an atomistic description of the mechanism for a simple prototype of helical membrane protein folding.

## Author summary

Many important cellular functions are performed by membrane proteins, and in particular by association of proteins via transmembrane helices. However, the mechanism of how the helices associate has been challenging to study, by either experiment or simulation. Here, we use advanced molecular simulation methods to overcome the slow time scales involved in helix association and dissociation and obtain a view of the association mechanism in atomic detail. We show that association occurs via an initially non-native dimer, before proceeding to the native state, and we validate our results by comparison to available experimental kinetic data. Our methods will also aid in the study of the assembly mechanism of larger transmembrane proteins via molecular simulation.

## Introduction

The folding of globular proteins has been extensively studied by experiment, theory and simulation, and can generally be described by a diffusive search on an energy landscape which is “funneled” toward the native state and minimally frustrated [1]. However, the folding of membrane proteins is less well explored, at least partially due to the greater difficulties of studying proteins folding in membranes in either experiment [2, 3] or simulation [4]. Membrane protein folding is nonetheless important to understand, because of the relevance of membrane protein misfolding to disease [5, 6]. Membrane proteins also constitute a significant fraction of all proteins (∼30%) in currently sequenced genomes, and represent an even higher fraction (over 60%) of drug targets [7, 8]. The folding of membrane proteins is quite distinct in nature from the folding of soluble, globular proteins, owing to the ordering effect of the lipid bilayer. In the case of transmembrane helical proteins, this effect has led to the proposal of a “two-stage model” [9, 10], in which the individual helices are first inserted into the membrane, before assembling to their native structure. In the cell, the analogous insertion process is usually accomplished by the translocon machinery [11, 12].

**Fig 1:**
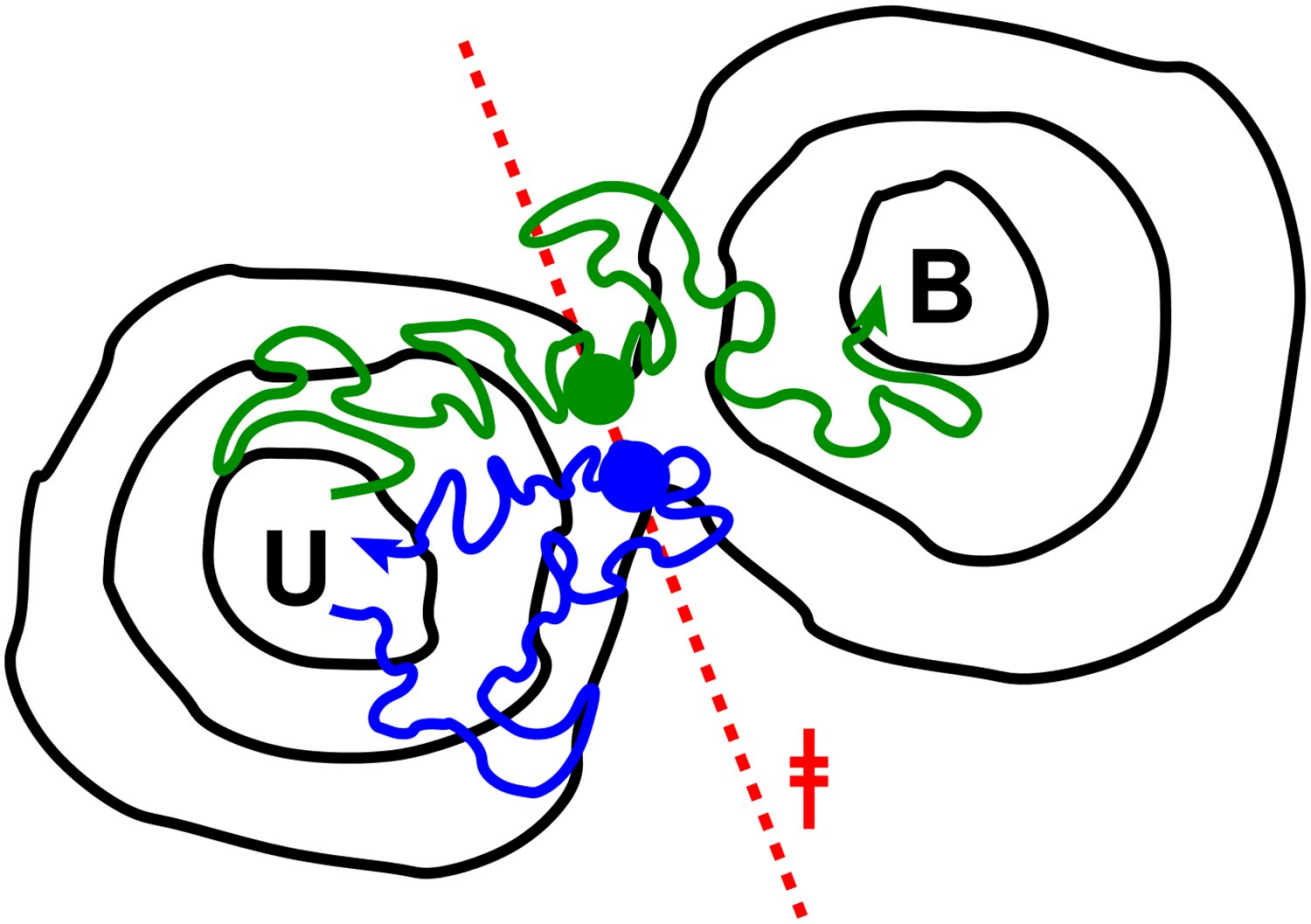
Diagram of transition-path sampling scheme used. A dividing surface (broken red line, ‡) is chosen as close as possible to the isocommitor (transition-state) surface on the free energy landscape (in the example shown we are interested in sampling transitions between the unbound and bound free energy minima, U and B, respectively). Sample configurations (blue, green circles) are chosen on this surface from umbrella sampling runs, and pairs of trajectories with conjugate momenta are launched from each (blue, green paths). In some cases, both forward and reverse trajectories end in the same basin (blue path), but in others they end in opposite basins and form a transition path (green path).

Simulations can play a vital role in helping to rationalize the folding and assembly mechanism of membrane proteins. Some elegant examples include the use of coarse-grained models to study the mechanism of GlpG folding [13], or to predict the topology of multi-pass transmembrane helical proteins [14]. Studying the details of the helix assembly into the specific native structure, however, requires a higher-resolution model. We have recently determined a free energy landscape for assembly of the glycophorin dimer, perhaps the quintessential example of transmembrane helix association, and the one which is the best-characterized [15–20]. The protein assembles into a parallel homodimer, in which the two helices dock via a GXXXG motif at the helix-helix interface (Fig 1) [21]. Despite being a paradigm for studies of helix association, the free energy of association had not been determined from simulations in a lipid bilayer until recently. In our study, we found that, while the native state was a local free energy minimum in the CHARMM 36 force field, it was unstable relative to the dissociated state [22]. However, this discrepancy could be resolved by a simple scaling of all the Lennard-Jones terms corresponding to protein-lipid interactions by a factor of 0.9, analogous to an approach used earlier for balancing protein-water [23] as well as protein-lipid [24] interactions. Here, we take the next step, and address the mechanism and kinetics of helix association in the membrane.

The viscosity of lipid membranes is much higher than water: for example, lateral self-diffusion coefficients for POPC in pure bilayers are ∼5 × 10^−8^ cm^2^s^−1^, [25] while peptides of similar molecular weight have translational diffusion coefficients of ∼3 × 10^−6^ cm^2^s^−1^ in water [26], i.e. in a given time interval their mean-square displacement in any given direction in water will be more than 50 times that for any lateral direction in a membrane. The combination of this viscosity with the energy barriers involved (in particular for dissociation) means that it is not feasible to study the association equilibrium of even a pair of single-pass transmembrane helices using long, unbiased simulations using commonly available resources. In our previous work, we employed umbrella sampling, using a carefully chosen reaction coordinate, in order to enhance sampling of binding [27] (although even so, this is very computationally demanding). While this is a robust method for obtaining equilibrium properties, it is not straightforward to determine binding and unbinding kinetics from umbrella sampling. Doing so requires an assumption for a dynamical model (e.g. one-dimensional diffusion) and a reliable method for estimating its parameters (e.g. position-dependent diffusion coefficients [28]) from the umbrella simulations, which is not trivial [29].

In this work, we use all-atom simulations with explicit membrane and water to study the assembly mechanism and kinetics of the glycophorin dimer. In order to overcome the above mentioned challenges due to the timescales involved and high membrane viscosity, we have turned to transition path sampling. In this way we greatly reduce the computational requirements, as only the trajectories between stable states need to be obtained, and the long waiting times in each stable state can be avoided. We determine that the initial association is dominated by non-native interactions, but that native interactions are key to formation of the correct dimer structure. Initial formation of non-native interactions is expected to enhance the overall rate of binding.

## Results and discussion

Here we take advantage of a transition path sampling (TPS) technique to capture the pathways and dynamics of glyophorin dimerization. The essential idea behind such schemes is that, while the waiting time to cross a free energy barrier may be extremely long, the time spent actually crossing the barrier can be many orders of magnitude smaller [30]. We use a variant of TPS in which a putative dividing surface *r*^‡^ is chosen on a reaction coordinate *r*, so that configurations *x* with *r*(*x*) = *r*^‡^ should (ideally) be close to the transition state for binding [31]. Configurations chosen from this dividing surface are then used to initialize pairs of trajectories with velocities of opposite sign; those pairs which end on opposite sides of the barrier are transition paths, and can be used to compute path properties (with appropriate weighting) and kinetics. The scheme is illustrated for reference in Fig 1. For efficiency, it is critical that the ensemble of configurations at the dividing surface should capture as closely as possible the true transition states. If they lie too far from the barrier, many pairs of trajectories will end in the same state and few transition paths will be found. For diffusive dynamics, the optimal fraction of transition paths obtained by this procedure should be 1/2.

**Fig 2:**
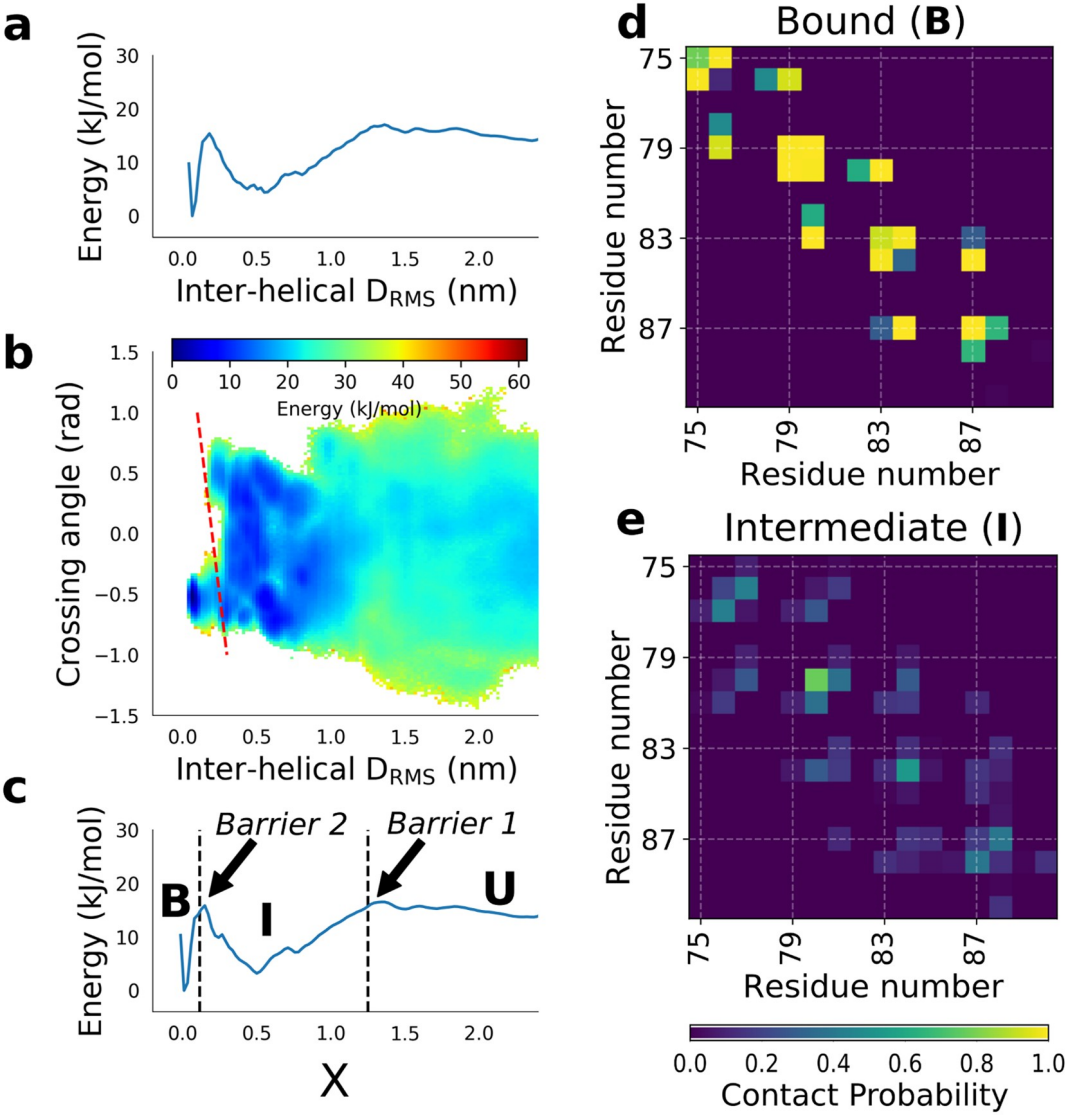
Binding free energy surfaces. (a) PMF projected onto the *D*_RMS_ coordinate used for original umbrella sampling. (b) 2D PMF projected onto helix-helix crossing angle and inter-helical *D*_RMS_. The approximate locus of the second barrier is indicated by a dashed red line. (c) PMF projected onto the hybrid collective variable *X*, with first and second barriers marked by black dashed lines. Contact maps for (d) the bound native state, B, and (e) bound non-native (intermediate) states, I.

We study glycophorin A dimerization using an all-atom force field (CHARMM 36) together with an explicit lipid bilayer and explicit solvent. We start with the equilibrium binding free energy surface determined in our earlier paper [22] for GpA dimerization, with the adjustment to protein-lipid interactions in the force field. This surface was determined by performing umbrella sampling simulations along the interhelical distance matrix RMS (*D*_RMS_, Eq 1) coordinate, a measure of the similarity of the interatomic distances in any given configuration to those in the native structure. This coordinate has previously proved effective for studying protein binding [22, 32], as it has similar features to the fraction of native contacts *Q* in protein folding [33], while remaining an effective bias coordinate once all intermolecular contacts are broken. The one-dimensional (1D) free energy surface on *D*_RMS_ in Fig 2a shows firstly that a stable intermediate is populated *en route* to the bound state. This intermediate is not visible in free energy surfaces computed for simple coordinates such as the helix-helix distance [22]. In the interests of efficiency, we have therefore divided our TPS into two steps [34], firstly the formation of the intermediate, and secondly conversion of the intermediate to bound, as described below. However, we first sought to obtain more insight into the nature of the bound, non-native intermediate, by computing the binding free energy surface as a function of two coordinates, *D*_RMS_ and the interhelical crossing angle *θ* (Fig 2b). *θ* is defined here as a pseudo-dihedral angle between the two helices [22], and is negative in the native state. This projection shows that the non-native minimum consists of an ensemble of conformations with similar *D*_RMS_ to native, but with both positive and negative helix-helix crossing angles, with an average crossing angle *θ* ≈ 0. Thus, in the intermediate, the helices do not have a strongly preferred orientation, being on average close to parallel, compared with the negative crossing angle found in the native state. Further insight into the nature of the intermediate can be obtained from the contact maps in Fig 2d and 2e. In contrast to the very well-defined contacts observed in the native state, a much broader set of residue-residue contacts are populated, suggestive of a more disordered state in which the two helices associate non-specifically in a roughly parallel orientation.

We also observed that while *D*_RMS_ is already a sufficiently good coordinate to discriminate non-native from native bound states, it is possible for the two helices to approach near-native values of *D*_RMS_ while still having the incorrect crossing angle. As discussed above, this would be highly detrimental to TPS efficiency, as selecting values of *D*_RMS_ close to the top of the apparent free energy barrier at *D*_RMS_ ≈ 0.2 nm would include many structures with an incorrect crossing angle which lie on the unbound side of the barrier. Although this has relatively little effect for the purposes of determining binding free energy, it is critical to the effectiveness of our TPS scheme.

We first noted that while neither the interhelical *D*_RMS_ nor the helix-helix crossing angle *θ* is by itself a good coordinate, their combination appears to better resolve both stable states and barriers. We therefore defined a new collective variable *X* as a linear combination of *D*_RMS_ and *θ* (Eq 2). The position of the new barrier on *X* is indicated by a dashed red line in Fig 2. The adjustable coefficient in the coordinate, *μ*, was picked so as to approximately maximize the free energy barrier between bound and unbound states projected onto *X* (see S1 Fig). The 1D potential of mean force *F*(*X*) is shown in Fig 2C. Two barriers along *X* were identified: the first barrier (between unbound and intermediate states) at *X*_‡,1_ = 1.25, and second barrier (between non-native bound intermediate and native state) at *X*_‡,2_ = 0.115. The positions of the barriers are indicated with thick dashed lines in Fig 2C.

We chose to perform separate transition path sampling simulations for each of the two barriers. This is because any transition path defined between the native dimer and fully dissociated state is expected, based on *F*(*X*), to spend the most time exploring the non-natively bound intermediate, and relatively little crossing barriers 1 and 2, which are the regions of interest for the mechanism. This would be very inefficient, defeating the purpose of TPS. Our division into two steps makes the assumption the equilibration within the intermediate should be fast relative to escape from it. From the umbrella sampling trajectories we therefore chose 75 frames on the dividing surface *X* = *X*_‡,1_ and 94 frames on the surface *X* = *X*_‡,2_, and we initiated pairs of trial trajectories with conjugate momenta from each. From these, we obtained 10 transition paths from barrier 1 and 25 transition paths from barrier 2. These rates of successfully sampling transition paths correspond, for barriers 1 and 2 respectively, to 21% and 67% of the theoretical maximum fraction of 50% reactive events for diffusive dynamics on an ideal reaction coordinate. The chosen reaction coordinate thus appears to be quite effective in locating transition states, although clearly not perfect. Note that most trajectory pairs terminated in reactant or product basins (S1 Table). Therefore, the chief reason for the unsuccessful trials was that both pairs of trajectories ended in the same basin (illustrated by blue trajectory in Fig 1) and were thus not valid transition paths.

**Fig 3:**
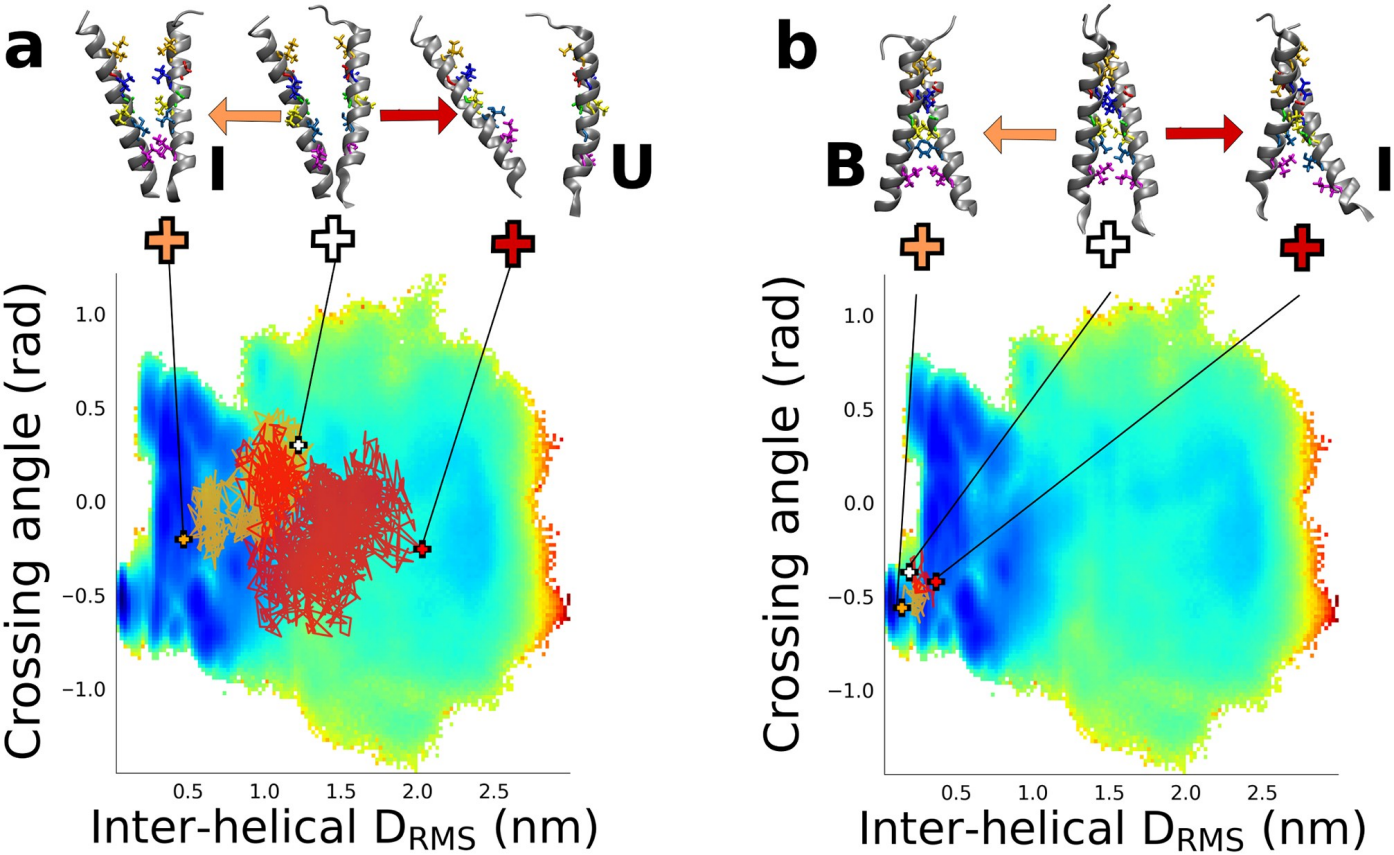
Transition paths for binding. (a) Transition paths for crossing the first barrier from U to I and projected onto the binding free energy surface. The “forward” and “reverse” halves of each trajectory are coloured light and dark brown respectively. Structures above plot illustrate the initial structure (center) and “forward” (left) and “reverse” (right) endpoints, ending in I and U respectively. Side-chains of key residues forming the native binding interface are shown in colour. (b) Same as for (a), but for crossing the second barrier from I to B.

Example transition paths projected onto the 2D free energy surface are shown in Fig 3. These transition paths are clearly of highly diffusive character for both transitions, with a large number of recrossings of the chosen dividing surfaces ‡_1_ and ‡_2_. This is likely a direct result of the very high viscosity of the membrane environment. Even the transition paths themselves may be rather long, particularly for the first barrier. Average transition-path durations were 570 and 4.6 ns for the first and second barriers, respectively (Full details of the transition path lengths are given in S1 Table). A descriptive picture of the binding may be obtained by examining snapshots drawn from transition paths characteristic of the two barrier crossings (Fig 3). The first step of binding starts with the separated peptides which encounter each other and form a stable intermediate via formation of (mostly) non-native interactions. In the second step, we see an initially non-native helix-helix interface first form the correct, in-register, native contacts before continuing to the fully formed native dimer structure.

**Fig 4:**
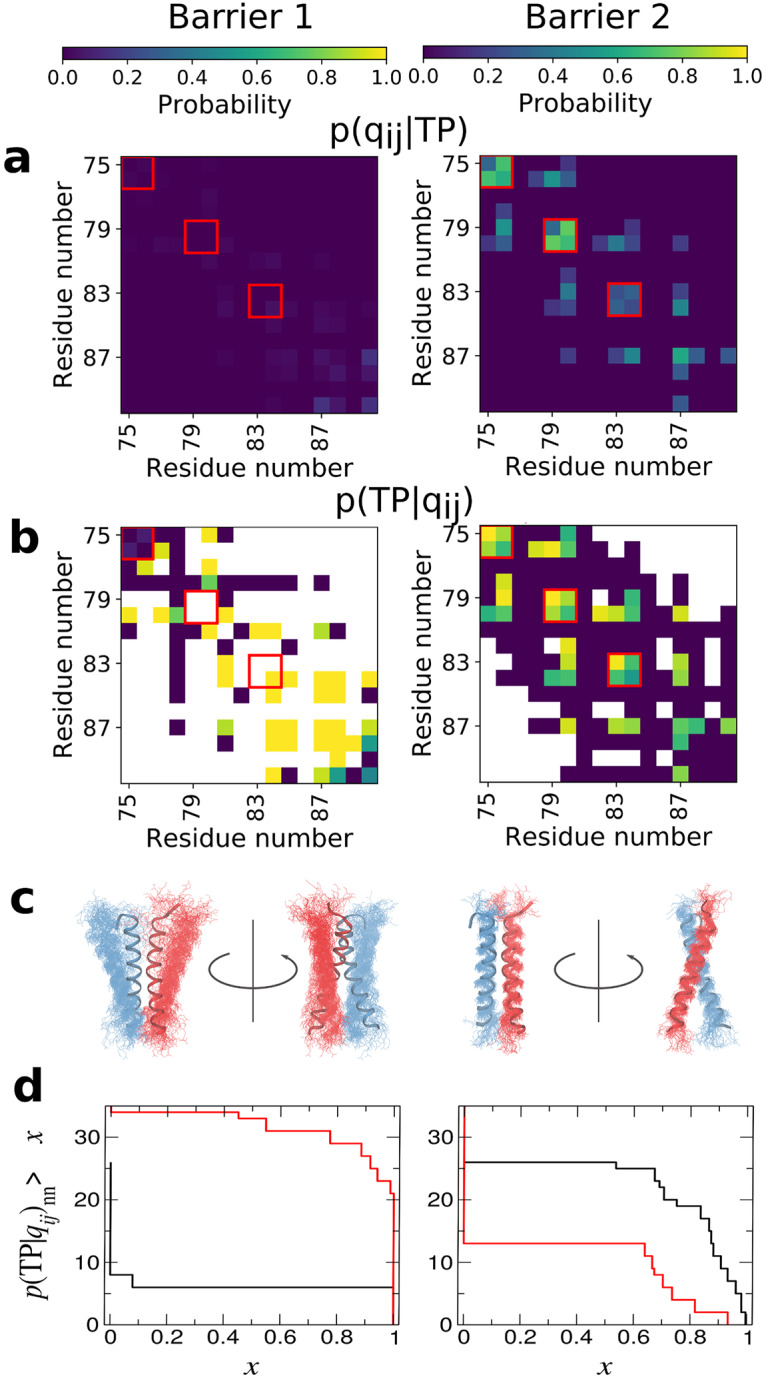
Characterizing the mechanism from transition paths. Left- and right-hand columns show first and second barriers, respectively. (a) Contact maps showing the probability a contact is formed in structures on transition paths. Key native contacts defining the GXXXG motif are indicated by red boxes. (b) Contact maps showing the conditional probability of being on a transition path given that a particular contact is formed. (c) Structural renders in cartoon representation of configurations at the barrier top, with chain a shown in blue and chain b shown in red. (d) Absolute tail distributions for *p*(TP|*q*_*ij*_)_nn_, i.e. the number of native contacts (black curves) and non-native contacts (red curves) which have *p*(TP|*q*_*ij*_)_nn_ greater than a give value. Note that the probability of forming any contact on a transition path for barrier 1 in (a) is very low, but the contacts which are predictive of transition paths in (b) are revealed after normalizing by the overall probabilities of contact formation.

To go beyond such an anecdotal description of the binding mechanism, we have analyzed the contacts formed on transition paths, which has proved to be a useful approach for the folding of globular proteins [35], even having a direct connection to experimental *ϕ*-values [36]. We first compute the average population of every possible residue-residue contact over all transition paths, that is *p*(*q*_*ij*_|TP), the probability that contact *q*_*ij*_ is formed, given that a snapshot is on a transition path (Fig 4a). A residue-residue contact is defined as any pair of heavy atoms, one from each residue, being within 0.45 nm. This shows that, on average, very few contacts are formed in crossing the first barrier, while native contacts appear to be formed in crossing the second barrier. None of this is surprising, given the above pictorial description of the binding. However, what we really would like to know is how *predictive* is the formation of a given contact of the binding reaction proceeding. To quantify this, we have calculated *p*(TP|*q*_*ij*_)_nn_, the probability of being on a transition path, given that a particular contact *q*_*ij*_ is formed, and that the snapshot is not part of the end (bound) state [35]. The results, shown in Fig 4b, yield a very clear picture of the mechanism. Many contacts are predictive of formation of the intermediate from the unbound, suggesting that formation of non-specific attractive interactions between the helices is sufficient to drive formation of the intermediate. However, the contacts which are most predictive tend to be localized towards the C-termini of the two peptides. This is consistent with the example shown in Fig 3 for this step. Notably, formation of contacts found in the native dimer structure is generally not predictive of initial formation of the non-native intermediate from unbound—which is in accord with the fact that there are many more ways to form an initial non-native complex than a native one. On the other hand, for the second step of converting the intermediate to the native state, the predictive contacts are essentially all native, or adjacent to native contacts, highlighting that formation of the native binding interface is crucial for this step. The important role for native contacts in mechanism is similar to what was found for globular proteins [35]. We have confirmed this quantitatively by computing absolute tail distributions of *p*(TP|*q*_*ij*_)_nn_, that is, the number of each type of contact for which *p*(TP|*q*_*ij*_)_nn_ is greater than a specified value (Fig 4d). These distributions show that indeed non-native contacts are more important for crossing the first barrier, while native contacts are crucial to crossing the second barrier.

In our simulations, we have used a force field where we reduced protein-lipid interactions by 5% via a small change to the Lennard-Jones combination rule. Because this adjustment is not specific to any particular contacts it is unlikely to change the overall mechanism. Linear free energy relationships would predict that this change would shift transition state 2 away from the native state towards the intermediate—which if anything strengthens our conclusions regarding the importance of native contacts in the mechanism.

From the transition-path sampling, we can also obtain estimates for the binding and unbinding rates for each step of the process. We obtain an off rate for barrier 2 of 2 × 10^3^ s^−1^ and an on rate of 3 × 10^4^ s^−1^, while on and off rates for barrier 1 are both approximately 10^7^ s^−1^. Converting the on-rate for barrier 1 to a bimolecular rate using concentration units of peptides per bilayer area (assuming an area of 0.76 nm^2^ per POPC lipid [18]), we obtain *k*_on_ ≈ 6 × 10^5^ molecule^−1^nm^2^s^−1^. Since the rates for the second barrier are so much faster, it is apparent that the first barrier will essentially control the steady-state association and dissociation rates.

Although experimental data for dimerization kinetics of glycophorin have not yet been reported, the folding kinetics of another transmembrane helix homodimer of a designed peptide, anti-*α*_IIb_, have been measured by Gai and co-workers [37]. This peptide has a similar dimerization interface to glycophorin, and a comparable dimerization affinity. Fluorescent probes were used to monitor binding, insertion and dimerization by fitting a kinetic model. This provides a first estimate for the association rate of a transmembrane helix dimer. Converting the reported rates to units of protein concentration per lipid area, we obtain an on rate of ∼150 molecule^−1^nm^2^s^−1^. This is two orders of magnitude slower than what we observe, but it is measured for a different peptide in vesicles with different lipid composition from the bilayer we study. In particular, anti-*α*_IIb_ has positively charged tags (KK) at each end to increase its water solubility, which are likely to interact electrostatically with the POPG molecules in the POPC/POPG vesicles in which it is studied, slowing diffusion, as well as repelling each other when the helices are close.

Although an experimental dissociation rate for glycophorin has not been reported in the literature, it has been estimated from the equilibration time in steric trap experiments to be on the order of hours [38]. In comparing this rate with that from simulation, we note that the dissociation constant in simulation is still significantly less favourable than estimated from steric trap experiments [22]. We compare with the steric trap experiments since they are also performed in POPC bilayers, and the *K*_*d*_ is known to be sensitive to the environment (e.g. bilayer versus detergent micelles [39–41]). Assuming that all of the difference in *K*_*d*_ comes from the off rate, we can correct the simulated off rate, to obtain a value of 2 × 10^−3^ s^−1^, within the same minute-hour range estimated experimentally (S1 Text).

Beyond agreement with wild-type rates, the mechanistic details of our simulations could also be validated experimentally if kinetic data for mutants were available—the effect of mutations on the rates could be used to compute folding *ϕ*-values, which can be directly related to the quantity *p*(TP|*q*_*ij*_) we have computed (Fig 4b), as shown previously [36].

### Conclusions

We find that dimerization of glycophorin occurs in two steps. In the first, the two peptides associate via non-native contacts, particularly towards the C-terminus, to form an initial non-native intermediate. Formation of an initial non-native complex (or encounter complex) is also a common step in protein-protein association in solution [42, 43]. Formation of an encounter complex with favourable free energy should in fact accelerate the association rate, as it helps the helices to remain in contact long enough to search for the native dimerization interface, rather than dissociating. In the second step, the non-native intermediate is converted to the native state via formation of the correct, native binding interface. This process is driven by native contact formation, analogous to the mechanism of folding of globular proteins.

Although glycophorin is the simplest example of helix association in the membrane, it is nonetheless yields important insights which should be helpful for future studies of the folding and assembly of larger helical transmembrane proteins. It seems likely that for larger systems would also have the potential for initial non-native helix docking before association into the native structure, driven by formation of specific native contacts. This could be tested by extending the approach presented here to more complex transmembrane proteins.

## Methods

### Force field and system setup

We study a dimer of the transmembrane region of glycophorin, residues 69-97 in a palmitoyl oleoylphosphatidylcholine (POPC) membrane with all-atom representation of both lipid, protein and solvent. We use the variant of the CHARMM 36 force field [44, 45] in which the protein-lipid interactions have been adjusted to stabilize protein-protein interactions in the membrane, based on glycophorin data [22]. Simulations were run with GROMACS version 4.6.7 at a constant temperature of 300 K with stochastic velocity rescaling [46] and pressure of 1 bar with a Parrinello-Rahman barostat [47]. Shifted Lennard-Jones interactions were cut off at 1.2 nm. Long-range Coulomb interactions were calculated with the Particle Mesh Ewald (PME) method [48], using a grid spacing of 0.12 nm and a real-space cut-off of 1.2 nm. Other detailsa are as previously described [22].

### Initial configurations for TPS

We performed replica exchange umbrella sampling (REUS) simulation to characterize the energetics of GpA dimerization [49]. The weighted histogram analysis method (WHAM) [50] was used to recover the unbiased free energy surface [27]. Two independent REUS simulations were performed: the first REUS simulation was started with all the windows initialized from the bound configuration (i.e. the experimental structure PDB id 1AFO [19]), and each umbrella window was run for 296 ns. The second REUS simulation was initialized with all windows in an unbound configuration, and run for 293 ns. These different initial conditions are referred to in this work as “start together” and “start separate”, respectively. The biasing was done along the interhelical distance root-mean-square *D*_RMS_ collective coordinate, defined as

$$\begin{array}{c} D_{\text{RMS}}={\left[N_{\text{nat}}^{-1}\sum_{(i,j)\in \text{nat}}{\left(r_{ij}-r_{ij,0}\right)}^{2}\right]}^{1/2} \end{array}$$ (1)

where the sum runs over the *N*_nat_ pairs of native contacts (*i*, *j*), *r*_*ij*_ is the distance between *i* and *j* in the conformation of interest and *r*_*ij*,0_ is the corresponding distance in the native structure. The native contacts in this case are intermolecular native distances between pairs of heavy atoms within *X* nm in the native structure, and within the residue range 78-88.

Since the PMFs from the two sets of REUS simulations were comparable [27], the data from both sets were pooled together in the construction of the unbiased PMF. We have projected the resulting PMF onto other coordinates, in order to identify hidden barriers. One such projection was done along the helix-helix crossing angle *θ* and the interhelical *D*_RMS_. The helix-helix crossing angle was defined as a pseudo-dihedral angle between the C*α* atoms of residues A78,A88,B88,B78 in that order, where A and B are the two chains of the dimer. The projection has revealed an existence of a hidden energy barrier along interhelical *D*_RMS_. We proposed a new collective variable “X” defined using the interhelical *D*_RMS_ and helix-helix crossing angle variables in the following way:

$$\begin{array}{c} X=D_{\text{RMS}}/\text{nm}+\mu \theta /\text{rad} \end{array}$$ (2)

The coefficient *μ* was determined to maximize the energy barrier between bound and unbound states, yielding a near-optimal value of 0.1 (see S1 Fig).

Two barriers along X were defined: the first barrier, between bound and unbound states, at *X* = 1.25 and the second barrier between bound native and bound non-native states at *X* = 0.115. From the REUS trajectories, frames within 0.005 of the barriers 1st and 2nd along X coordinate were selected. A random 39 frames were selected from the “start together” REUS simulation, and further 36 frames were randomly from the “start separate” REUS run for the first barrier, with the corresponding numbers for the second barrier being 45 and 49.

Each frame was used to initialize a pair of trajectories constituting a transition path sampling run. A “forward” trajectory was initialized with random velocities chosen from a Maxwell-Boltzmann distribution, and a “backward” trajectory was initialized with same random velocities, only with the opposite sign.

### Basin definitions for TPS

A transition path sampling run starts in the proximity of the barrier peak. The run terminates when the simulation enters one of two user-defined basins, or when target simulation time is reached, whichever comes earlier. Transition paths are those pairs of trajectories which end in different basins.

The basins ware defined as intervals along the X collective variable, by inspecting the PMF: for the 1st barrier the basins were defined as BasinA(-0.5—0.07) and BasinB(0.4—2.5). For the 2nd barrier the basins were defined as BasinA(-0.5—0.4) and BasinB(2.1—2.5).

### Calculation of rates and weights from TPS

We computed rates from the transition path trajectories following the method proposed by Hummer [31]. Briefly, for each attempted transition path, we save the velocity *v* = *dX*/*dt* each time it crosses the initial value of *X* from which all the trajectories were launched. Then we estimate the rate of binding (*k*_*b*_) and unbinding (*k*_*u*_) from

$$\begin{array}{c} \frac{2}{k_{b}^{-1}+k_{u}^{-1}}=p_{eq}\left(X^{‡}\right)\langle \theta_{\text{TP}}{\left(\sum_{i}{\left|v_{i}\right|}^{-1}\right)}^{-1}\rangle \end{array}$$ (3)

where *p*_*eq*_(*X*^‡^) is the probability density at *X*^‡^, *θ*_TP_ is 1 for succesful transition paths and 0 otherwise, *v*_*i*_ is the velocity of crossing the initial surface during the *i*’th crossing in a given transition path attempt, and the average is taken over shooting attempts from the initial surface *X*^‡^. The binding and unbinding rates are separated using the equilibrium constant determined by integration of the potential of mean force *F*(*X*).

Note that in all the above TPS calculations, attention is always restricted to the two states whose interconversion is being considered, rendering it essentially a two state problem. That is all probabilities and probability densities are calculated by excluding the third state not being considered in each case, and transition path probability densities are for one barrier only.

For calculation of average contact maps and transition path lengths, each transition path was weighted according to the weighting scheme [31]:

$$\begin{array}{c} w_{i}\propto {\left(\sum_{i}{\left|v_{i}\right|}^{-1}\right)}^{-1} \end{array}$$ (4)

This corrects for the fact that the sampling method used is biased towards trajectories which frequently recross the initial surface.

### Calculation of *p*(TP|*q*_*ij*_)_nn_

We computed the probability of being on a transition path given that the dimer was not already native, *p*(TP|*q*_*ij*_)_nn_, as

$$\begin{array}{c} p{\left(TP|q_{ij}\right)}_{\text{nn}}=\frac{p\left(q_{ij}|TP\right)p{\left(TP\right)}_{\text{nn}}}{p{\left(q_{ij}\right)}_{\text{nn}}} \end{array}$$ (5)

where *p*(*q*_*ij*_|*TP*) is the probability of contact *q*_*ij*_ being on transition paths, which is obtained directly from the statistics of contact formation on the obtained transition paths, and *p*(*q*_*ij*_)_nn_ is the probability of a contact *q*_*ij*_ being formed in any non-native state (i.e. not bound state), obtained from umbrella sampling. The third quantity, *p*(TP)_nn_, the probability of being on a transition path if not in the bound state, is obtained from

$$\begin{array}{c} p{\left(TP\right)}_{\text{nn}}=\frac{2\tau_{\text{TP}}}{2\tau_{\text{TP}}+k_{\text{bind}}^{-1}} \end{array}$$ (6)

where *τ*_TP_ is the mean transition path duration and *k*_bind_ is the binding rate determined from transition path sampling.

## Supporting information

S1 Text Details of adjusting simulation off-rates to account for the difference between stability in experiment and simulation. (PDF)

S1 Table Summary of details of shooting attempts in transition-path sampling simulations. (PDF)

S1 Fig Plot of the dependence of the apparent barrier height for dimerization as a function of the adjustable parameter λ defining the reaction coordinate. (TIFF)
