# Supplementary material for: Atomistic mechanism of transmembrane helix association
Source: PLoS Comput Biol. 2020 Jun 4;16(6):e1007919. doi: 10.1371/journal.pcbi.1007919 (PMC7272003; doi:10.1371/journal.pcbi.1007919)
Supplement: S1 Text — (PDF) [file pcbi.1007919.s001.pdf]

S1 Text: Comparison of off rate with steric trap experiments

The off rate from simulations was estimated to be 2×10^3 s^{-1}. However, even with the force field adjustment to stabilize the native state, the dimer is somewhat less stable than in experiment, having $K_d \approx 10^{-3}$ molecules.nm^{-2} ($\Delta G \approx 4.1$ kcal.mol^{-1}), compared with the esimate from steric trap experiments of $K_d \approx 1.3{\times}10^{-9}$ molecules.nm^{-2} ($\Delta G \approx 12.1$ kcal.mol^{-1}) [1]. If we assume that the on rate $k_{\rm on}$ is unaffected by changes in stability, since it is effectively diffusion-limited, then any change of stability or dissociation constant $K_d = k_{\rm off}/k_{\rm on}$ must arise from variations in the off rate $k_{\rm off}$. Thus, since K_d is 10^6 smaller in experiment, $k_{\rm off}$ would be expected to be 10^6 smaller also, i.e. 2×10^{-3} s^{-1}.

References

[1] Hong H, Blois TM, Cao Z, Bowie JU. Method to measure strong protein-protein interactions in lipid bilayers using a steric trap. Proc Natl Acad Sci USA. 2010 Nov;107(46):19802–19807.
