# Supplementary material for: Atomistic mechanism of transmembrane helix association
Source: PLoS Comput Biol. 2020 Jun 4;16(6):e1007919. doi: 10.1371/journal.pcbi.1007919 (PMC7272003; doi:10.1371/journal.pcbi.1007919)
Supplement: S1 Table — (PDF) [file pcbi.1007919.s002.pdf]

S1 Table : Summary of all the transition path sampling simulation executed. Each row in the table corresponds to a forward and backward pair of simulations. The "barrier" refers to the first or second barrier in the PMF, the "initial condition" is that used to initialize the umbrella sampling runs from which the staring structure was chosen, "window" refers to the umbrella window from which that structure was drawn, and "simulation frame" is the frame number in that window corresponding to that structure. The final column lists the end points of the two trajectories, which may be "A" or "B" for finishing in the more native or less native side of the barrier respectively, and "None" if the trajectory did not reach either basin within the maximum simulation time allowed. Path durations listed as 0.0 are those paths which were shorter than the interval between saving frames.

Barrier	Initial Condition	Fwd basin	Fwd duration (ns)	Rev basin	Rev duration (ns)
1st	separate	B	16.3	B	21.2
1st	separate	B	42.0	B	877.6
1st	separate	B	100.7	B	19.4
1st	separate	B	62.8	B	30.5
1st	separate	B	73.5	B	356.3
1st	separate	None	-	A	587.3
1st	separate	B	296.9	None	-
1st	separate	B	129.6	B	115.1
1st	separate	None	-	None	-
1st	separate	B	13.0	B	1,176.1
1st	separate	A	975.5	B	716.4
1st	separate	B	1,301.5	A	627.4
1st	separate	A	411.0	A	735.0
1st	separate	B	939.8	B	954.3
1st	separate	None	-	B	192.4
1st	separate	A	612.5	B	237.7
1st	separate	B	731.8	B	493.3
1st	separate	B	413.1	B	134.1
1st	separate	B	290.2	B	637.7
1st	separate	B	480.0	None	-
1st	separate	B	82.5	B	193.3
1st	separate	B	270.9	B	775.3
1st	separate	None	-	None	-
1st	separate	B	207.8	B	401.5
1st	separate	None	-	B	117.2
1st	separate	None	-	B	540.8
1st	separate	B	647.5	B	113.5
1st	separate	B	91.0	B	13.8
1st	separate	None	-	B	129.0
1st	separate	B	181.2	B	336.0

Continued on next page

S1 Table – continued from previous page

Barrier	Initial Condition	Fwd basin	Fwd duration (ns)	Rev basin	Rev duration (ns)
1st	separate	B	67.3	B	39.9
1st	separate	B	604.8	None	-
1st	separate	B	1,031.3	None	-
1st	separate	B	758.3	B	929.0
1st	separate	B	282.7	None	-
1st	separate	B	216.2	A	1,059.9
1st	native	B	755.2	B	718.0
1st	native	B	444.8	B	756.0
1st	native	None	-	None	-
1st	native	None	-	None	-
1st	native	B	816.2	B	981.9
1st	native	None	-	B	120.2
1st	native	B	636.1	B	173.0
1st	native	A	865.6	None	-
1st	native	B	191.5	B	89.4
1st	native	B	230.4	B	47.0
1st	native	B	437.9	B	96.6
1st	native	None	-	None	-
1st	native	B	403.0	B	418.9
1st	native	A	168.3	B	308.6
1st	native	B	740.5	B	185.2
1st	native	None	-	A	293.1
1st	native	B	169.3	None	-
1st	native	None	-	None	-
1st	native	B	203.2	None	-
1st	native	B	136.7	A	227.0
1st	native	A	127.9	B	311.6
1st	native	None	-	B	556.8
1st	native	B	449.0	B	77.3
1st	native	B	263.7	B	443.8
1st	native	B	442.7	None	-
1st	native	None	-	B	229.8
1st	native	B	150.9	B	40.7
1st	native	B	552.5	B	329.5
1st	native	B	1,255.0	A	664.7
1st	native	B	359.1	B	556.5
1st	native	None	-	None	-
1st	native	B	21.4	B	11.4
1st	native	None	-	None	-
1st	native	B	186.8	B	175.7
1st	native	B	267.1	None	-
1st	native	A	270.5	B	107.3

Continued on next page

S1 Table – continued from previous page

Barrier	Initial Condition	Fwd basin	Fwd duration (ns)	Rev basin	Rev duration (ns)
1st	native	None	-	B	135.7
1st	native	B	315.4	B	180.3
1st	native	A	1,178.4	B	308.5
2nd	separate	A	0.0	A	0.1
2nd	separate	A	0.3	A	33.2
2nd	separate	A	1.3	A	12.7
2nd	separate	A	0.6	A	0.2
2nd	separate	A	0.1	A	0.8
2nd	separate	A	2.0	B	3.2
2nd	separate	B	37.4	A	0.1
2nd	separate	A	8.0	A	0.1
2nd	separate	A	6.6	B	30.8
2nd	separate	A	0.8	A	5.7
2nd	separate	B	5.3	A	9.7
2nd	separate	B	10.6	A	15.4
2nd	separate	A	11.2	B	57.5
2nd	separate	None	-	A	0.6
2nd	separate	B	42.5	B	10.6
2nd	separate	B	6.5	B	45.7
2nd	separate	B	3.1	A	123.4
2nd	separate	A	0.2	A	0.0
2nd	separate	B	4.8	B	36.9
2nd	separate	B	9.5	A	8.9
2nd	separate	A	0.0	A	0.9
2nd	separate	B	11.8	A	6.0
2nd	separate	A	0.1	A	0.0
2nd	separate	A	3.0	A	3.3
2nd	separate	A	0.1	A	2.8
2nd	separate	A	2.8	A	2.5
2nd	separate	A	0.1	A	0.7
2nd	separate	B	118.7	B	94.3
2nd	separate	A	11.3	B	35.7
2nd	separate	A	0.1	A	2.7
2nd	separate	A	4.4	A	4.6
2nd	separate	A	1.1	A	0.2
2nd	separate	A	26.6	A	1.7
2nd	separate	A	0.0	A	0.1
2nd	separate	A	2.3	A	11.0
2nd	separate	A	6.4	A	2.0
2nd	separate	A	0.5	A	3.3
2nd	separate	A	3.3	A	1.7
2nd	separate	A	0.2	A	1.0

Continued on next page

S1 Table – continued from previous page

Barrier	Initial Condition	Fwd basin	Fwd duration (ns)	Rev basin	Rev duration (ns)
2nd	separate	A	1.3	A	2.7
2nd	separate	A	0.0	A	0.2
2nd	separate	A	13.1	B	7.4
2nd	separate	A	0.5	B	4.0
2nd	separate	A	0.1	A	0.2
2nd	separate	B	6.3	A	0.8
2nd	separate	A	0.9	A	9.1
2nd	separate	A	0.1	B	44.6
2nd	separate	A	0.3	A	0.2
2nd	separate	A	0.3	A	0.7
2nd	native	A	40.6	A	109.5
2nd	native	A	21.8	B	54.1
2nd	native	B	19.4	A	16.0
2nd	native	B	65.5	A	0.2
2nd	native	A	0.2	A	0.2
2nd	native	A	3.4	A	9.2
2nd	native	A	7.6	A	4.1
2nd	native	A	97.2	B	100.9
2nd	native	A	1.4	A	3.0
2nd	native	A	7.9	A	18.7
2nd	native	A	16.9	B	132.2
2nd	native	A	4.7	A	6.8
2nd	native	A	26.5	A	56.7
2nd	native	A	141.0	A	42.0
2nd	native	B	66.4	A	8.0
2nd	native	A	2.9	A	0.2
2nd	native	A	127.0	A	45.9
2nd	native	A	168.7	A	115.5
2nd	native	B	103.0	A	19.5
2nd	native	A	0.2	A	1.5
2nd	native	A	0.4	A	0.4
2nd	native	A	0.9	A	2.2
2nd	native	A	0.3	A	0.4
2nd	native	B	0.4	A	0.0
2nd	native	B	60.1	A	0.0
2nd	native	A	104.7	A	132.4
2nd	native	B	15.0	B	22.4
2nd	native	B	19.7	A	3.4
2nd	native	A	7.9	A	1.5
2nd	native	A	0.0	A	0.0
2nd	native	A	0.5	A	4.2
2nd	native	A	0.0	A	0.1

Continued on next page

S1 Table – continued from previous page

Barrier	Initial Condition	Fwd basin	Fwd duration (ns)	Rev basin	Rev duration (ns)
2nd	native	A	0.0	A	0.0
2nd	native	A	0.0	A	0.0
2nd	native	A	7.4	A	0.7
2nd	native	A	1.1	A	1.4
2nd	native	A	80.6	None	-
2nd	native	A	0.1	A	0.5
2nd	native	B	62.3	B	30.3
2nd	native	B	2.7	B	3.2
2nd	native	A	44.4	A	9.4
2nd	native	None	-	A	4.7
2nd	native	A	7.0	B	16.2
2nd	native	B	4.5	B	2.1
2nd	native	A	107.1	None	-
