# Supplementary figures and images for: Atomistic mechanism of transmembrane helix association
Source: PLoS Comput Biol. 2020 Jun 4;16(6):e1007919. doi: 10.1371/journal.pcbi.1007919 (PMC7272003; doi:10.1371/journal.pcbi.1007919)

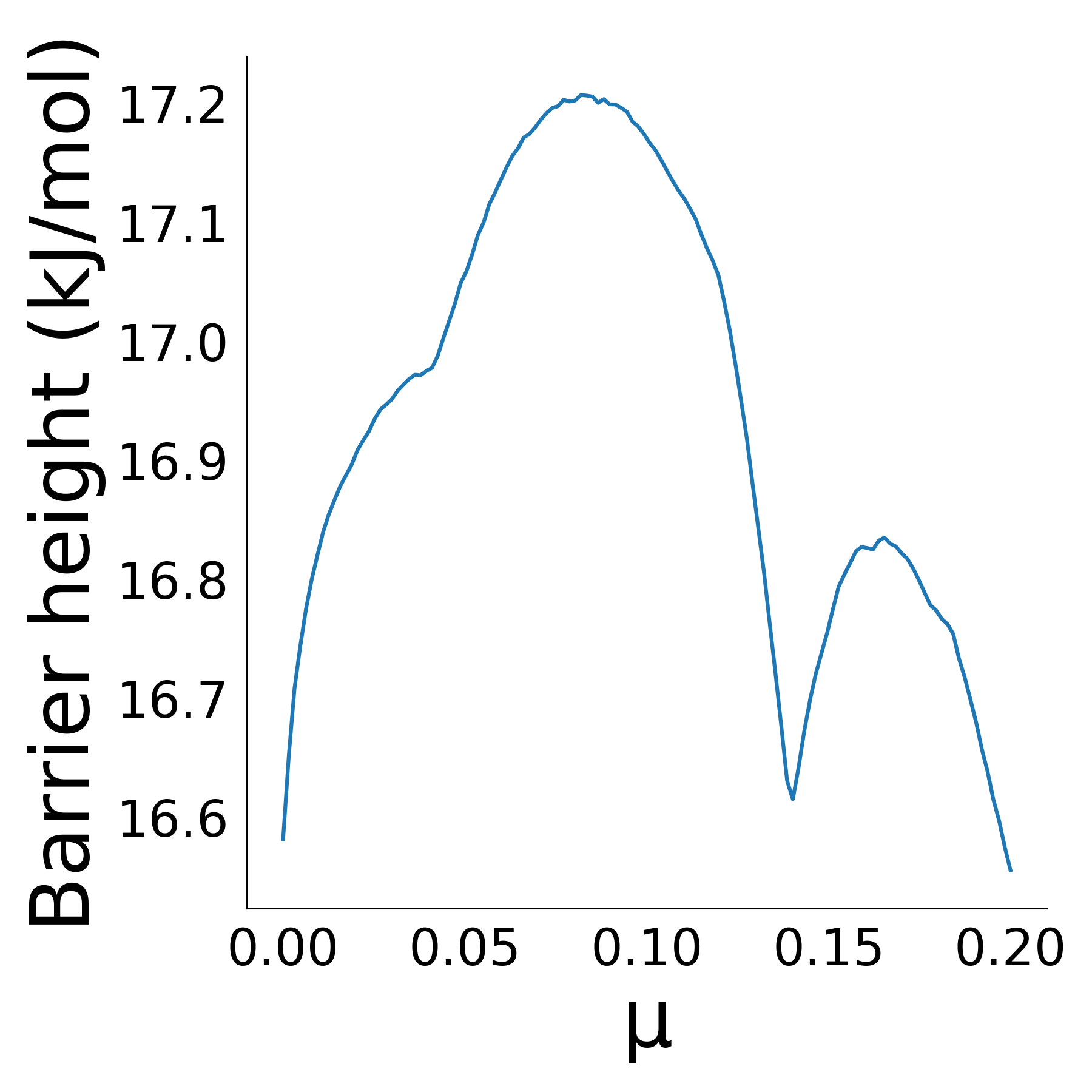

Supplement: S1 Fig — (TIFF) [file pcbi.1007919.s003.tiff]
